# Editorial Comment: Epidemiology of Spinal Cord Injury in Adults in Sweden, 2016-2020: A Retrospective Registry-Based Study

**DOI:** 10.1590/S1677-5538.IBJU.2025.9909

**Published:** 2025-02-15

**Authors:** Charlotta Josefson, Tiina Rekand, Åsa Lundgren-Nilsson, Katharina S. Sunnerhagen

**Affiliations:** 1 Gothenburg University Institute of Neuroscience and Physiology Department of Clinical Neuroscience, Sahlgrenska Academy Gothenburg Sweden Department of Clinical Neuroscience, Sahlgrenska Academy, Institute of Neuroscience and Physiology, Gothenburg University, Gothenburg, Sweden;; 2 Haukeland University Hospital Department of Neurology Bergen Norway Department of Neurology, Haukeland University Hospital, Bergen, Norway

**Neuroepidemiology. 2024 Aug 13:1-9 Online ahead of print.**

DOI: 10.1159/000540818 | ACCESS: 39137737

## COMMENT

This is a retrospective registry-based study over 4 years at the National Quality Register for Rehabilitation Medicine in Sweden, which included 26 units around the country and evaluated the epidemiological characteristics of the Swedish spinal cord injury (SCI) population from January 2016 to December 2020. The data analyzed were gender, age, etiology, level of injury, neurogenic bowel, bladder dysfunction, complications during rehabilitation and the need for positive airway pressure or ventilator. Mean age was 56 years (male = 66%). Tetraplegia was more common among traumatic SCI (TSCI) than non-traumatic SCI (NTSCI). The incidence was 11.9–14.8 per million for TSCI and 8.9–11.8 per million for NTSCI. At discharge, 8% of patients needed a breathing aid. Of those who were ventilator-dependent at discharge, 75% had a TSCI. Disturbed bowel and bladder functioning were noted in 58% of patients at discharge. The median time spent at the unit was 40 days, but it was approximately 2 weeks longer for those with a TSCI.

SCI is a major cause of long-term disability, with a higher prevalence among males (1,2). The main complications among these patients were pyelonephritis (14.5% of TSCI cases and 4.5% of NTSCI cases) and pressure ulcers (10.2% of TSCI cases and 3.3% of NTSCI cases). Sweden has a lower incidence of SCI and its complications in comparison to Western Europe and globally (3), maybe due to increased awareness initiatives. With falls being the primary cause of TSCI and a high age at beginning, the Swedish SCI population has a pattern resembling that of other Scandinavian countries.
